# Supplementary material for: Backmapping triangulated surfaces to coarse-grained membrane models
Source: Nat Commun. 2020 May 8;11:2296. doi: 10.1038/s41467-020-16094-y (PMC7210967; doi:10.1038/s41467-020-16094-y)
Supplement: Supplementary file 1 — Supporting Information [file 41467_2020_16094_MOESM1_ESM.pdf]

Supplementary Information

Backmapping triangulated surfaces to coarse-grained membrane models

Pezeshkian et al,

Supplementary Note

TS2CG: Triangulated Surfaces to Coarse Grained Models. TS2CG is implemented in C++ and includes two separate scripts. Pointillism and CG Membrane Builder. The code will continue to be available as part of the Martini tool-box on github and will be further developed (https://www.github.com/marrink-lab/TS2CG). The planned development includes optimization of the user interface and implementation of adaptations required for selecting regions and making these compliant with PBC. Interested users are welcome to join us in the development.

Pointillism: The script is compiled into an executable binary file with the name PLM that reads a DTS simulation trajectory file or a triangulated surface file and performs step 1 and 2. The output file contains vertex positions, area, principal curvatures, normal vector and principal direction. The script requires a triangulated surfaces file and several parameters as arguments in the command line (see user manual, available in the source code folder). A triangulated surface file that can be read by this script should have an extension of ".q" or ".dat". The *.q file is formatted as shown below.

```
50   50   50
1840
0       21.4    33.8     32.7    0
1       38.1    26.1     32.3    0
2       40.9    24.2     19.9     0
.
.
.
1839    31.2   323.2    23       0
0
3680
0   75    776     1043     1
1   796   1821    752      1
2   995   1027    279      1
3   662   1162    56       1
4   167   38      391      1
.
.
```

Line 1: Box information (3 double numbers).
Line 2: Number of vertices (NV, 1 integer number).
Line 3 to NV+2: Vertex ID and coordinate (1 integer number and 3 double numbers).
Line NV+3: Number of triangles (NT, 1 integer number).
Line NV+4 to NV+NT+3: Triangle ID and ID of its vertices (4 integer number).

The *.dat file additionally contains information about the position of the proteins and is described in the user manual. Please note: using *.q file, proteins can be placed at specific positions (see user manual).

CG membrane builder: The script is compiled into an executable binary file with the name PCG that performs step 3 and 4 from the Pointillism output files and generates a Gromacs based coordinate file. If the system contains protein, or if the user wants to add proteins, a Gromacs coordinate file (.gro file format) of the protein structure should be provided. By default, Martini forcefield lipids will be used. To backmap to another CG forcefield a lipid structure library should be provided. This script requires a few parameters that are read by arguments from command line and a user-friendly input file (with *.str extension). The input file should contain information about desired lipid type at each monolayer, lipid type number

ratio, area per lipid and Gromacs based coordinate file name of the proteins. For more details see the user manual.

Note: TS2CG can also be used to generate large scale complex membranes with different shapes for any CG simulations by providing a proper TS structure and a CG model lipid library (Supplementary Figure 4). To build an all-atom structure, a two steps backmapping scheme can be used, in which we first backmap a DTS output to a CG Martini model and then, after equilibration, the CG structure can be backmapped to an all-atom representation.

Supplementary Methods

Obtaining the geodesic connecting two neighboring vertices: Consider two vertices (v_{-1}, v_1) that are connected by a vector link $\vec{\mathbf{l}}$ of a size of l. Our calculation is performed in a coordinate in which $\vec{\mathbf{l}}$ is the $\mathbf{x}$ axis, and the origin is the middle point between (v_{-1}, v_1)(Figure 2-B main manuscript). A transformation matrix that takes a point in the global coordinate to this coordinate can be obtained using Householder transformation. The projection of $\vec{\mathbf{l}}$ on each vertex plane (P_{-1}, P_1) are denoted as $\mathbf{m}_{-1}, \mathbf{m}_1$ (Figure 2-B main manuscript). A geodesic curve that connects these two vertices is given by a parametric representation as $\vec{\boldsymbol{s}}_{\mathrm{G}}(t) = l/2(t, Y(t), Z(t))$. Note: for our purpose, we only need to evaluate $\vec{\boldsymbol{s}}_{\mathrm{G}}(0)$. We inquire $\vec{\boldsymbol{s}}_{\mathrm{G}}(t)$ to satisfy 3 conditions on each vertex (6 conditions in total).

1) It should cross both vertices

$$\vec{\boldsymbol{s}}_{\mathrm{G}}(0) = \frac{l}{2}(i, 0,0), \qquad i = -1,1 \qquad (1)$$

2) $\vec{\mathbf{R}}(t)$, must be parallel to $\mathbf{m}_{-1}, \mathbf{m}_1$ when it crosses v_{-1}, v_1 respectively.

$$\vec{\boldsymbol{s}}_{\mathrm{G}}{}'(i) = \widetilde{\mathbf{m}}_i \ , \qquad i = -1,1 \qquad (2)$$

where $\widetilde{\mathbf{m}}_{-1}$, $\widetilde{\mathbf{m}}_1$ are given as

$$\widetilde{\mathbf{m}}_i = \frac{l}{2}\frac{\mathbf{m}_i}{m_{i,\mathrm{x}}} = \frac{l}{2}\left(1, \frac{m_{\mathrm{y},i}}{m_{\mathrm{x},i}}, \frac{m_{\mathrm{z},i}}{m_{\mathrm{x},i}}\right), \quad i = -1,1 \qquad (3)$$

3) $\vec{\boldsymbol{s}}_{\mathrm{G}}$ curvature at v_i points must be equal to the curvature of the v_i in the direction of $\widetilde{\mathbf{m}}_i$. This condition is not as trivial to fulfill. We will convert this boundary condition to a condition on $\vec{\boldsymbol{s}}_{\mathrm{G}}{}''(i)$ as below.

Curvature of v_i in the direction of $\widetilde{\mathbf{m}}_i$ can be obtained using Euler curvature formula

$$C_i = C_1 \cos^2(\theta_i) + C_2 \cos^2(\theta_i) \qquad (4)$$

where C_1, C_2 are principal curvatures at v_i and θ_i is the angle between $\boldsymbol{m}_i$ and main principal direction $(\hat{\mathbf{e}}_1(v_i))$ at v_i.

The tangent vector at each point of the curve can be found as

$$\widehat{\mathbf{T}} = \frac{\frac{d\widehat{\mathbf{T}}}{dt}}{\left|\frac{d\widehat{\mathbf{T}}}{dt}\right|} = \frac{l}{2} \times \frac{\widehat{\boldsymbol{x}} + Y_t(t)\widehat{\boldsymbol{y}} + Z_t(t)\widehat{\boldsymbol{z}}}{\sqrt{1 + Y_t^2(t) + Z_t^2(t)}} \qquad (5)$$

where $Y_t(t)$ and $Z_t(t)$ are derivate of $Y(t)$ and $Z(t)$ with respect to t. Curvature of $\vec{\boldsymbol{s}}_{\mathrm{G}}(t)$, C, can be found as

$$C\hat{\mathbf{N}} = -\frac{d\hat{\mathbf{T}}}{dS} \qquad (6)$$

where

$$dS = \frac{l}{2}\sqrt{1 + Y_t^2(t) + Z_t^2(t)}\, dt \qquad (7)$$

By performing a few strength forward analytical steps, a relationship between curvature and first and second derivatives of $\vec{\boldsymbol{s}}_{\mathrm{G}}(t)$can be obtained as

$$C(t)\hat{\mathbf{N}}(t) = -\frac{l}{2}\times\left\{\frac{Y_{tt}(t)\hat{\mathbf{y}} + Z_{tt}(t)\hat{\mathbf{z}}}{1 + Y_t^2(t) + Z_t^2(t)} - \frac{Y_{tt}(t)Y_t(t) + Z_{tt}(t)Z_t(t)}{\left(1 + Y_t^2(t) + Z_t^2(t)\right)^{\frac{3}{2}}}\hat{\mathbf{T}}(t)\right\} \qquad (8)$$

where C is the curvature of the curve at t.

For $i = -1,1$, $Y_t(i) = l\widetilde{m}_{\mathrm{y},i}/2$ and $Z_t(i) = l\widetilde{m}_{\mathrm{z},i}/2$ (Supplementary Equation 3), and $\hat{\mathbf{N}}(i)$ is known (normal vector to the plane of the vertex). Substituting these in Supplementary Equation 8 and solving it with respect to $Y_{tt}(i)$ and $Z_{tt}(i)$, we find

$$Y_{tt}(i) = \frac{2C_i|\widetilde{\mathbf{m}}_i|^2}{l}\left(N_{\mathrm{x},i}\left(\frac{2\widetilde{m}_{\mathrm{y},i}}{l}\right) - N_{\mathrm{y},i}\right) \qquad (9)$$

$$Z_{tt}(i) = \frac{2C_i|\widetilde{\mathbf{m}}_i|^2}{l}\left(N_{\mathrm{x},i}\left(\frac{2\widetilde{m}_{\mathrm{z},i}}{l}\right) - N_{\mathrm{z},i}\right) \qquad (10)$$

To satisfy these 6 boundary conditions, $(Y(t), Z(t))$, must be at least a polynomial of degree of 5

$$\begin{cases} Y(t) = \sum_{n=0}^{n=5} a_n t^n \\ Z(t) = \sum_{n=0}^{n=5} b_n t^n \end{cases} \qquad (11)$$

which gives

$$Y(0) = a_0 = \frac{Y_{tt}(1) + Y_{tt}(-1) + 5Y_t(-1) - 5Y_t(1)}{16} \qquad (12)$$

$$Z(0) = b_0 = \frac{Z_{tt}(1) + Z_{tt}(-1) + 5Z_t(-1) - 5Z_t(1)}{16} \qquad (13)$$

Implementation of the boundary condition 3 is difficult. We have observed that, generating an extended TS by only satisfying condition 1 and 2 gives a very close structure to the above procedure. In this case

$$Y(0) = a_0 = \frac{Y_t(-1) - Y_t(1)}{4} \qquad (14)$$

$$Z(0) = b_0 = \frac{Z_t(-1) - Z_t(1)}{4} \qquad (15)$$

Therefore, for different applications one can use Supplementary Equation 14 and 15.

Vesicle Growth in DTS simulation: The DTS simulation was performed on a closed triangulated surface containing 1060 vertices. The system energy is described as

$$E_{\mathrm{b}} = \frac{\kappa}{2}\sum_{1}^{N_{\upsilon}}(2H_{\upsilon})^2 A_{\upsilon} + \frac{K_{\mathrm{v}}}{2V_{\mathrm{f}}}(V - V_{\mathrm{f}})^2 + \frac{k_{\mathrm{r}}}{8h^2 A_{\mathrm{f}}}(\Delta A - \Delta A_{\mathrm{f}})^2 \qquad (16)$$

and

$$\Delta A = 2h\sum_{\upsilon}^{N_{\upsilon}} 2H_{\upsilon}A_{\upsilon} \qquad (17)$$

where $2h$ is the membrane thickness and k_{r} is the area compression modulus.[1] In this simulation, we have chosen the membrane bending rigidity $\kappa = 20k_{\mathrm{B}}T$, $K_{\mathrm{v}} = 60k_{\mathrm{B}}T$, $k_{\mathrm{r}} = 3\kappa = 60k_{\mathrm{B}}T$, $\Delta A_{\mathrm{f}} = 0.3A_{\mathrm{i}}$, $V_{\mathrm{f}} = 0.7\Upsilon(A_0)$ and $\Upsilon(A_0)$ is volume of a spherical vesicle with an area of A_0.

Considering $d = 4.5\mathrm{nm}$ (see the main text), the above input data is corresponding to following situations. Upon absorption, the area of the vesicle increases from $A_{\mathrm{i}} = 25446\mathrm{nm}^2$ to $A_{\mathrm{f}} = 32157\mathrm{nm}^2$. The processes happen quickly, so the volume of the vesicle remains constant ($V_{\mathrm{i}} = V_{\mathrm{f}}$; See main text). Assuming that the initial vesicle has a spherical shape, we find.

$$V_{\mathrm{f}} = \frac{4\pi}{3}\left(\frac{A_{\mathrm{i}}}{4\pi}\right)^{3/2} \qquad (18)$$

We assume that the absorption increases the mismatch between the upper and inner monolayer area from $\Delta A_{\mathrm{i}} = 4297\mathrm{nm}^2$ (a spherical vesicle) to $\Delta A_{\mathrm{f}} = 6544\mathrm{nm}^2$.

Supplementary Tables

System	DOPC	Gb3	STxB	Solvent (Anti-freeze WF)	Ion	FF	Time	Ensemble
Vesicle	92169	0	0	0	0	Dry Martini	200ns	NVT
Bud formation I	110791	1125	75	0	375 Na^{+}	Dry Martini	200ns	NPT
Bud formation II	54095	1125	75	7990264 (900000)	150 mmol	Wet Martini 2.2	50ns	NPT

Supplementary Table 1: details of the simulated systems.

Out Bilayer				
Inner Mono	POPC	SAPE	SAPI	CHOL
Mol #	190	69	69	28
APL	0.65	0.65	0.71	0.42
Outer Mono	POPC	SAPE	SAPI	CHOL
Mol #	190	138	0	28
APL	0.64	0.64	0	0.4
In Bilayer				
Inner Mono	POPC	POPE	SAPI	CRDL
Mol #	103	103	34	56
APL	0.67	0.66	0.72	1.1
Outer Mono	POPC	POPE	SAPI	CRDL
Mol #	172	138	0	18
APL	0.63	0.66	0	1.08

Supplementary Table 2: APL for different lipids in different lipid compositions.

Supplementary Figures

Figures were generated using VMD[2] (1.9.4a12) and power point (16.16.11). The graphs were plotted using gnuplot (5.2) and xmgrace (5.1.25).

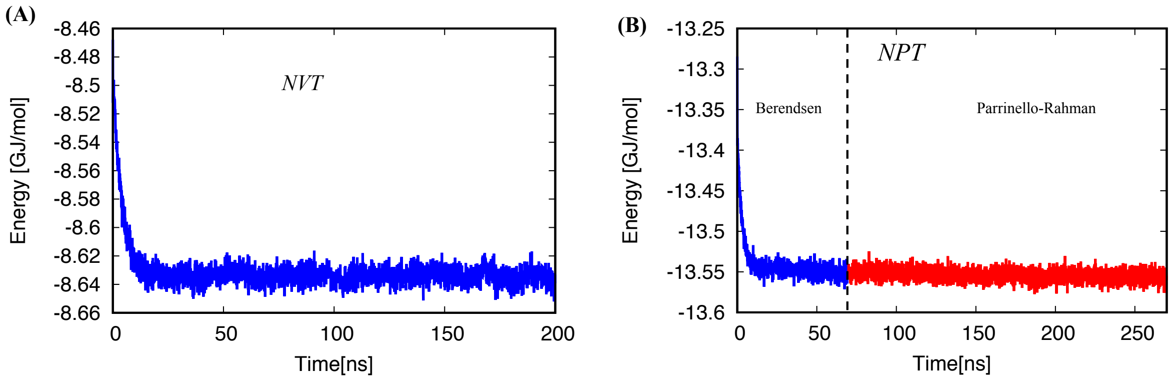

Supplementary Figure 1) (A) Total energy evolution of the deformed vesicle in Figure 3-C form the final 200ns. (B) Total energy evolution of the vesicular bud in Figure 4-B form 70ns NVT and 200ns NPT simulations.

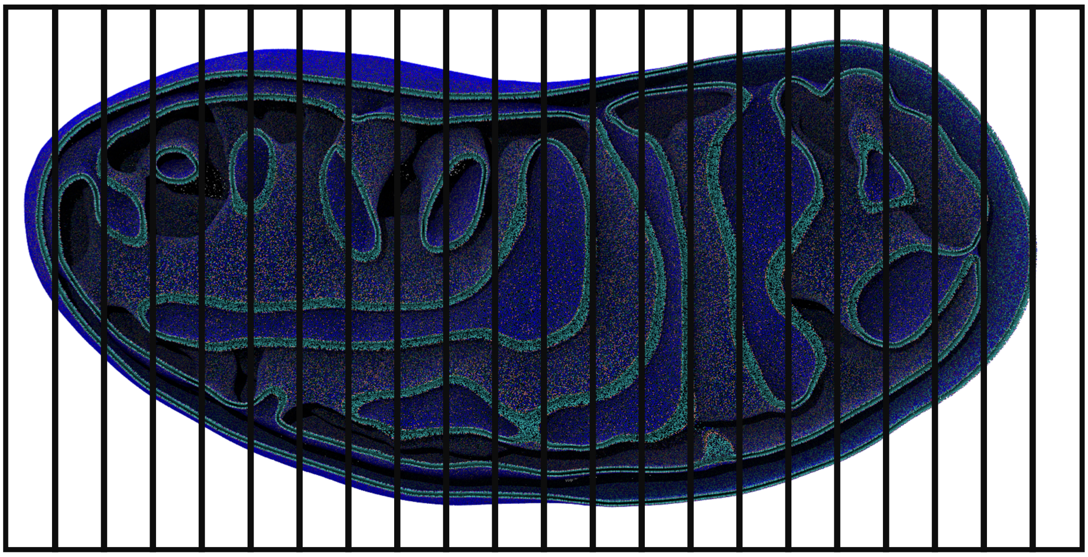

Supplementary Figure 2): The backmmaped structure was divided into 20 subsystems for faster and easier energy minimalization.

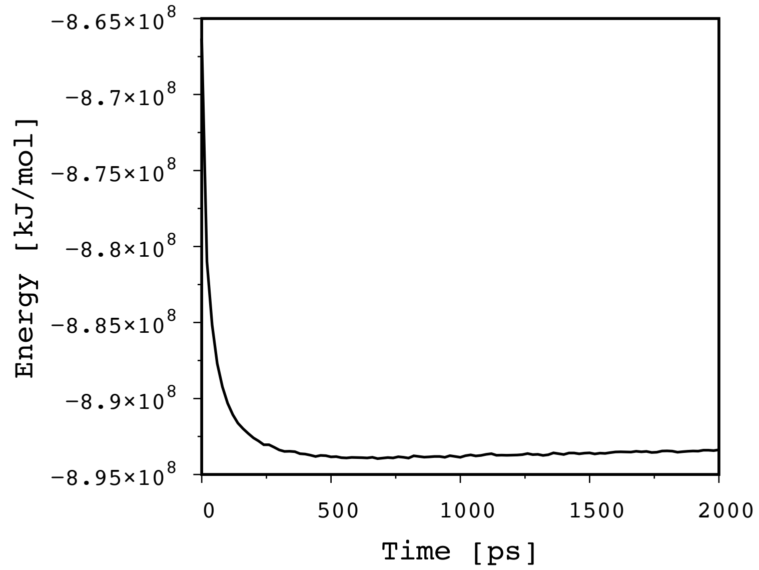

Supplementary Figure 3) Total energy evolution of the mitochondrion model during 2ns MD simulation.

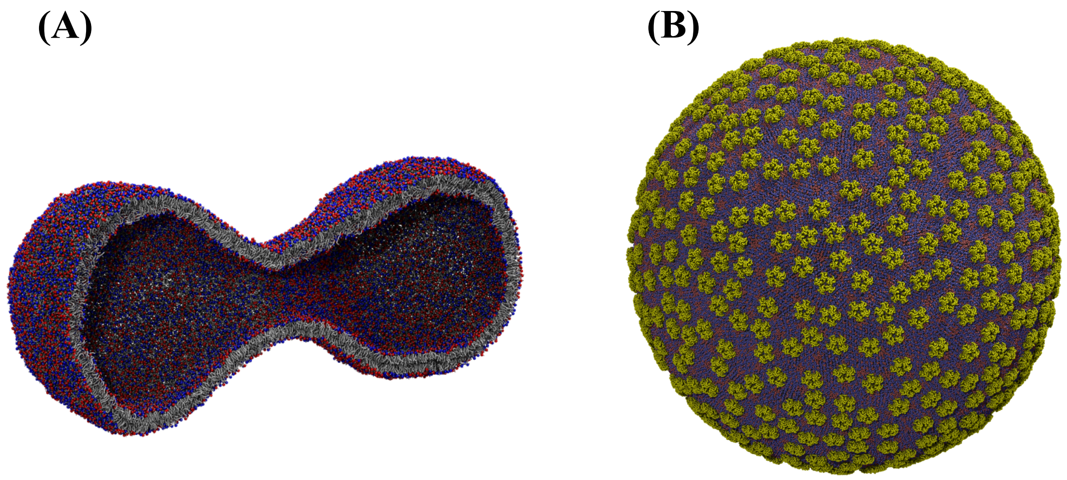

Supplementary Figure 4) (A) a deformed vesicle containing DPPC and POPC lipid mixture. (B) A crowded vesicle, with 50 percent of the surface area covered by peripheral membrane proteins.

Supplementary References

1 Seifert, U., Berndl, K. & Lipowsky, R. Shape transformations of vesicles: Phase diagram for spontaneous- curvature and bilayer-coupling models. *Phys Rev A* **44**, 1182-1202 (1991).

2 Humphrey, W., Dalke, A. & Schulten, K. VMD: visual molecular dynamics. *J Mol Graph* **14**, 33-38, 27-38, doi:10.1016/0263-7855(96)00018-5 (1996).
